# Supplementary material for: Mosquito salivary sialokinin reduces monocyte activation and chikungunya virus-induced inflammation via neurokinin receptors
Source: Nat Commun. 2025 Oct 20;16:8644. doi: 10.1038/s41467-025-64468-x (PMC12537910; doi:10.1038/s41467-025-64468-x)
Supplement: Supplementary file 1 — Supplementary Information [file 41467_2025_64468_MOESM1_ESM.pdf]

a

Aedes aegypti

Sialokinin I:

Asn–Thr–Gly–**Asp**–Lys–**Phe**–Tyr–**Gly**–**Leu**–**Met**–**NH$_2$**

Sialokinin II:

Asp–Thr–Gly–**Asp**–Lys–**Phe**–Tyr–**Gly**–**Leu**–**Met**–**NH$_2$**

Human

Substance P:

Arg–Pro–Lys–Pro–Gln–Gln–**Phe**–Phe–**Gly**–**Leu**–**Met**–**NH$_2$**

Neurokinin A:

His–Lys–Thr–**Asp**–Ser–**Phe**–Val–**Gly**–**Leu**–**Met**–**NH$_2$**

Neurokinin B:

Asp–Met–His–**Asp**–Phe–**Phe**–Val–**Gly**–**Leu**–**Met**–**NH$_2$**

b

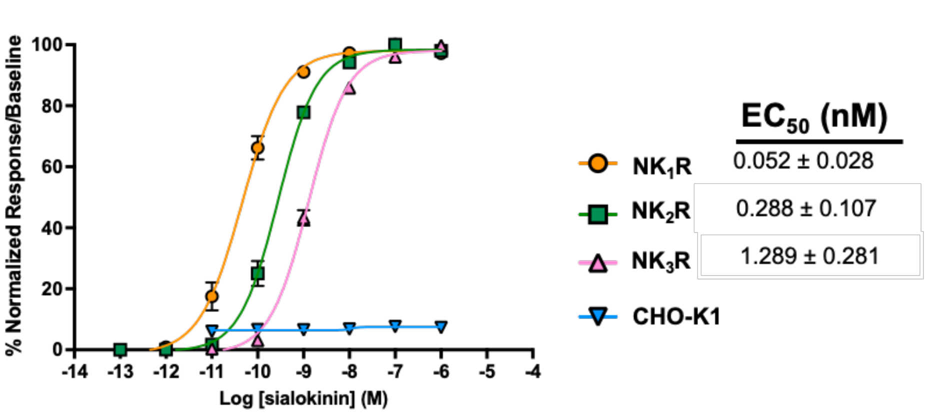

c

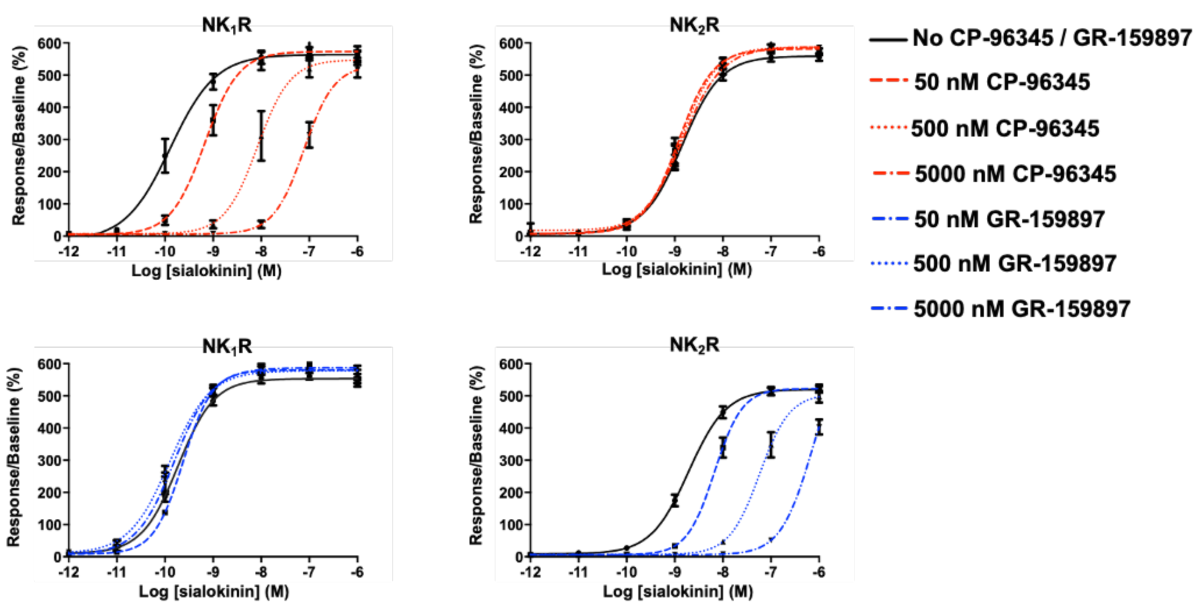

Supplementary Fig. 1: Molecular properties of sialokinin and its binding affinities to human neurokinin (NK) receptors. a, Sequence comparison of A. aegypti sialokinins and human tachykinins. Amino acid residues common to sialokinins are in boldface type. **b**, Dose response curve of sialokinin I interacting with NK receptors (NK1R, NK2R, and NK3R) expressed on Chinese hamster ovary (CHO) cells and un-transfected CHO (CHO-K1) cells. Results represent the mean ± SEM of three independent experiments run in triplicate. **c**, CP-96345 and GR-159897 specifically antagonize sialokinin-stimulated calcium influx in CHO cells expressing human NK receptors NK1R or NK2R. Cumulative concentration-effect curves were constructed for sialokinin in the absence or presence of CP-96345 or GR-159897 (at 50, 500, or 5000 nM concentrations). The control curve represents the response curves constructed on a non-CP-96345/GR-159897-treated preparation in each experiment. Data shown are mean ± S.E.M.

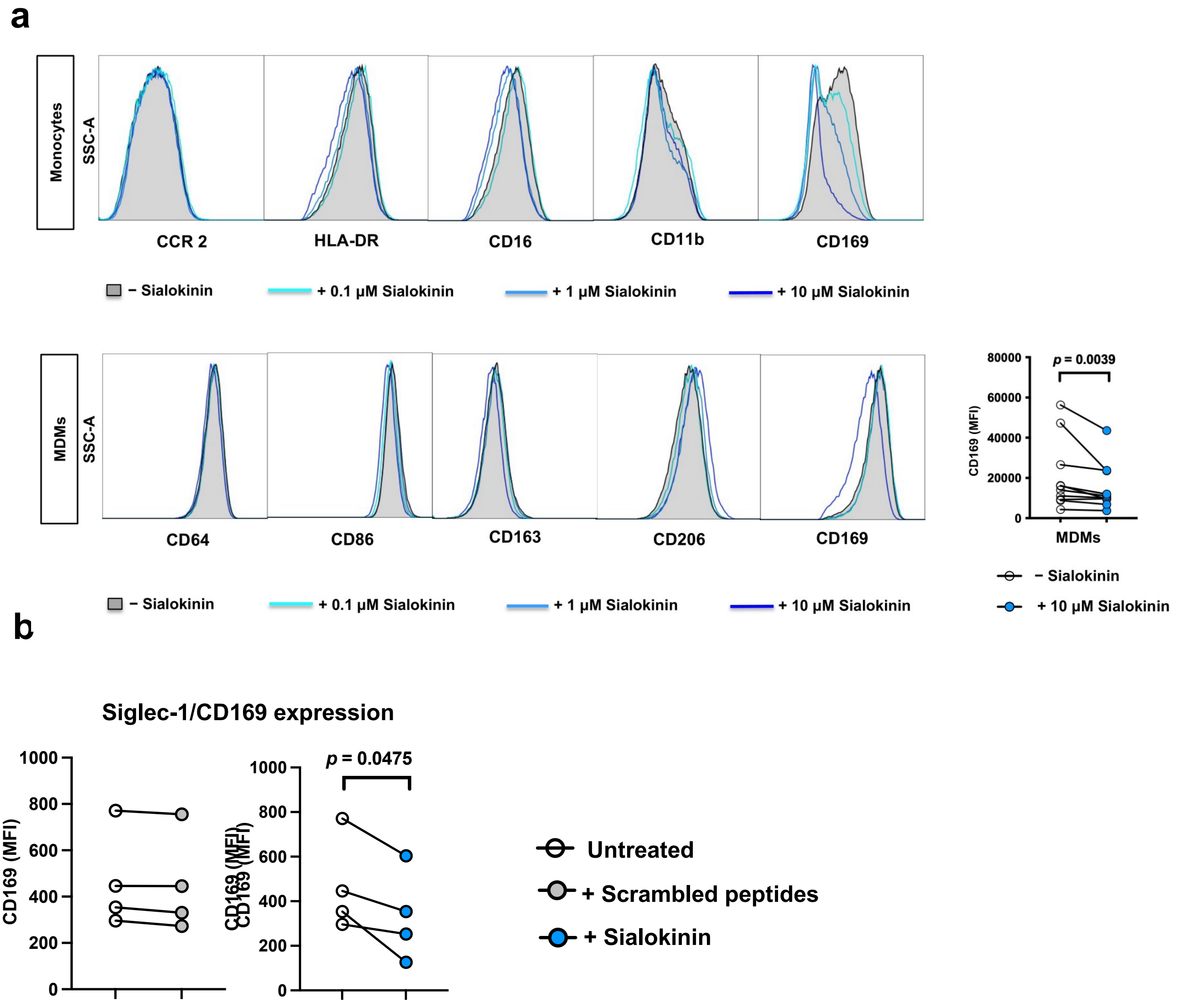

Supplementary Fig. 2: Effect of *Aedes aegypti* sialokinin peptides on human monocytes and macrophages. **a,** Sialokinin peptide modulates myeloid cell activation. Human monocytes and MDMs were treated with increasing concentrations (0.1, 1, and 10 µM) of sialokinin, and flow cytometry was performed to assess activation status of the cells following treatment. Siglec1/CD169 expression was reduced in sialokinin-treated monocytes and MDMs compared with untreated cells, with the greatest reduction seen after treatment with 10 µM sialokinin. Experiments were performed with cells isolated from ten donors. Values represent the mean fluorescence intensity (MFI) of CD169 expression of monocytes or MDMs. $^{**}p < 0.01$ by paired two-tailed *t*-test. **b,** Human monocytes were treated with scrambled peptide or sialokinin at 10 µM. Flow cytometry was performed to assess activation status of the cells following treatment. Siglec1/CD169 expression was reduced in monocytes treated with sialokinin but not in those treated with scrambled peptides. Experiments were performed with cells isolated from four donors. Values represent the mean fluorescence intensity (MFI) of CD169 expression of monocytes. $^{*}p < 0.05$ by paired two-tailed *t*-test.

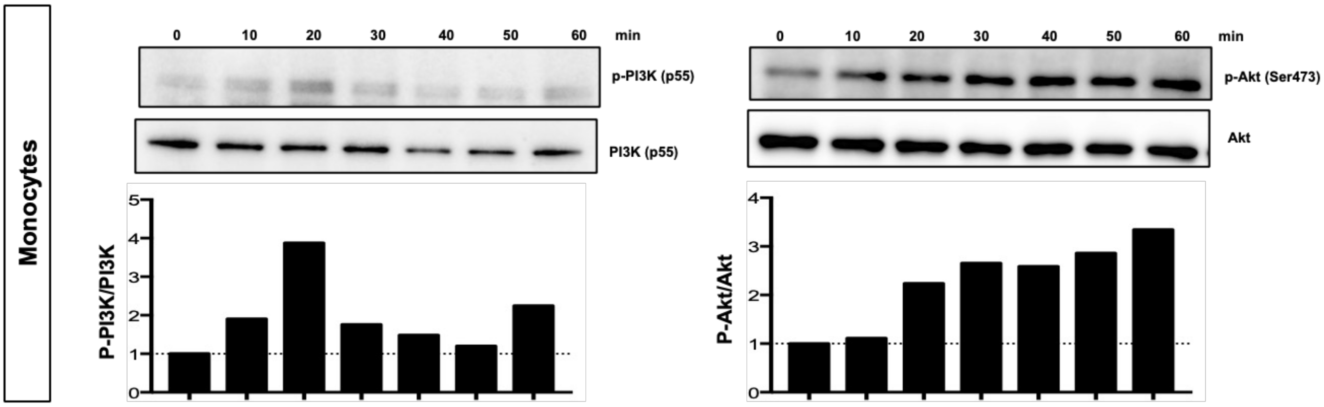

Supplementary Fig. 3: Sialokinin activates the PI3K/Akt signaling pathway in human primary monocytes. Western blot analyses show sialokinin-stimulated activation of PI3K and Akt in human monocytes. Levels of phosphorylated PI3K (P-PI3K)/PI3K and P-Akt/Akt were measured in the same blots.

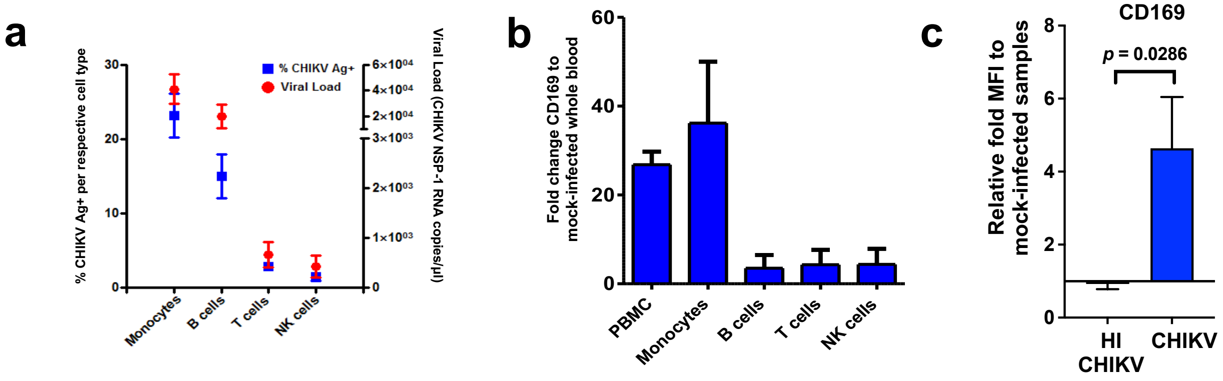

Supplementary Fig. 4: CHIKV infection in human whole blood. Whole blood was obtained and infected with heat-inactivated (HI) CHIKV or infectious CHIKV at a multiplicity of infection (MOI) of 10 for 24 hours. **a,** Detection of CHIKV nsP2 antigen and CHIKV viral load quantification in respective immune cell subsets by anti-CHIKV nsP2 antibody and NSP1 qRT-PCR (n = 3). **b,** CD169 gene expression analysis in respective immune cell subsets (n = 3). **c,** Fold geometric MFI change of CD169 in HI-CHIKV- or CHIKV-treated monocytes relative to mock-treated monocytes (n = 4). Data are presented as mean ± SD. $^{**}p < 0.01$ by two-tailed *t*-test.

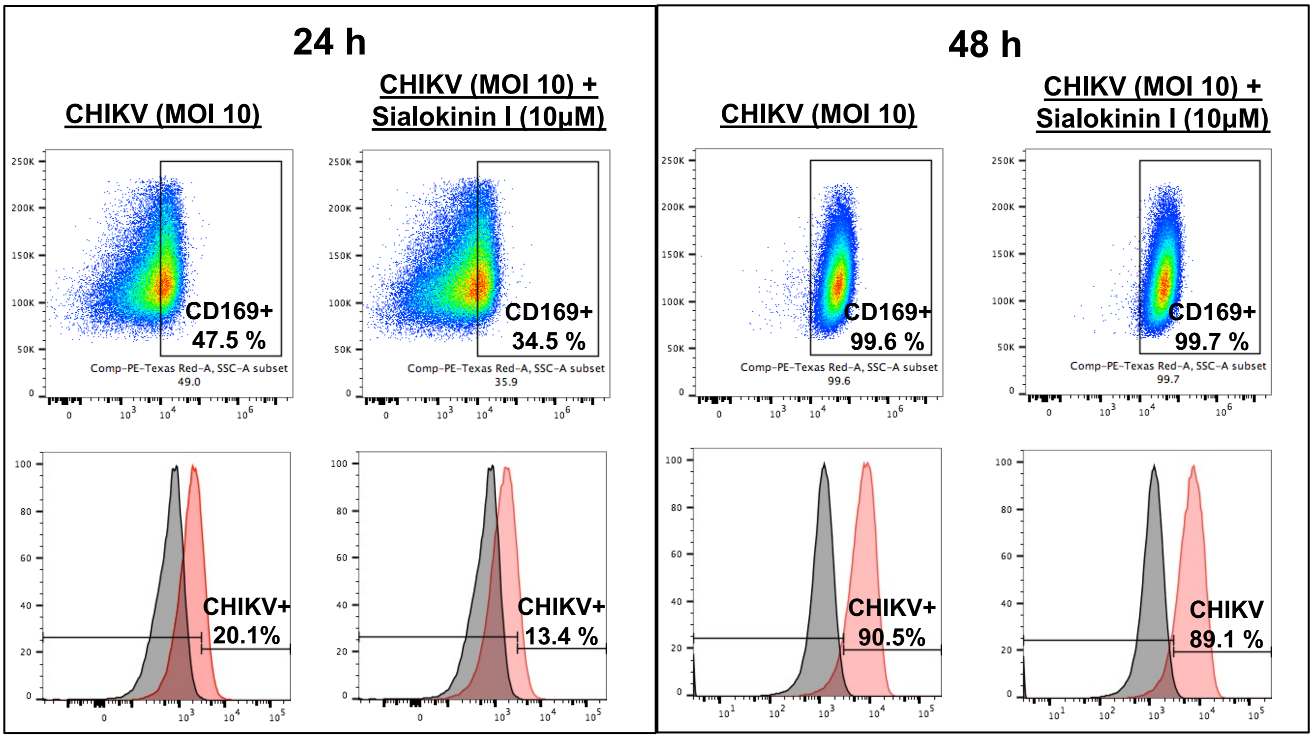

Supplementary Fig. 5: Effect of sialokinin on human monocyte activation and CHIKV infection at 24 and 48 hours post-infection. Human monocytes from healthy donor were infected with CHIKV in the presence or absence of sialokinin (10 µM). Activation profiles of monocytes were assessed by flow cytometry. CD169 expression was lower in sialokinin-treated cells following CHIKV infection (MOI = 10) at 24 hours post-infection. CHIKV infectivity in monocytes was reduced in the presence of sialokinin.

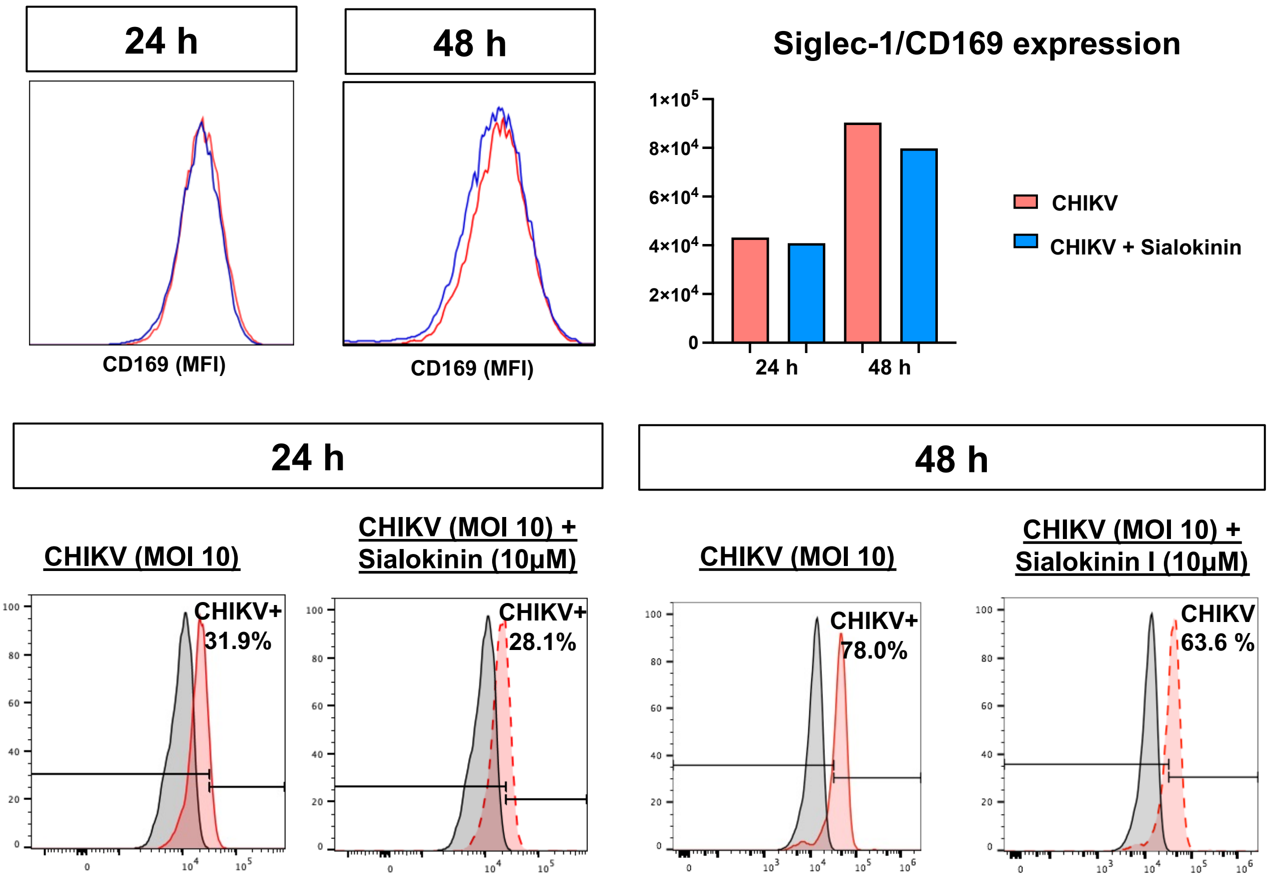

Supplementary Fig. 6: Effect of sialokinin on human monocyte-derived macrophages (MDMs) activation and CHIKV infection at 24 and 48 hours post-infection. Human MDMs from healthy donor were infected with CHIKV in the presence or absence of sialokinin (10 µM). Activation profiles of monocytes were assessed by flow cytometry. CD169 expression was lower in sialokinin-treated cells following CHIKV infection (MOI = 10) at 24 hours post-infection. CHIKV infectivity in MDMs was reduced in the presence of sialokinin.

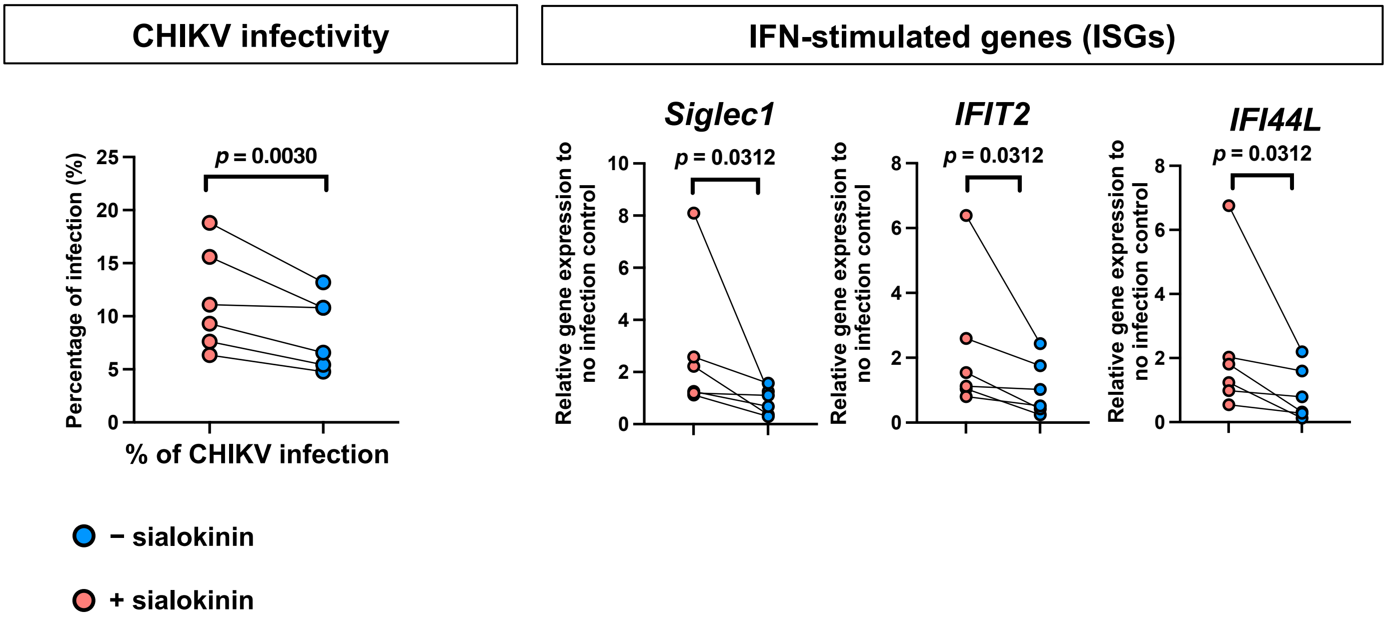

Supplementary Fig. 7: Sialokinin treatment attenuates CHIKV infection and suppresses IFN-stimulated gene expression in human monocytes at 24 hours post-infection. Primary human monocytes isolated from six healthy donors were treated with sialokinin (10 μM) and infected with chikungunya virus (CHIKV) at a multiplicity of infection (MOI) of 10. At 24 hours post-infection, intracellular CHIKV levels were quantified by flow cytometry. Total RNA was extracted from harvested cells, and quantitative real-time PCR was performed to assess mRNA expression of interferon-stimulated genes (ISGs), including *Siglec1*, *IFIT2*, and *IFI44L*. Relative gene expression was calculated using the ΔΔCt method, normalized to housekeeping gene, and expressed relative to the uninfected control group. Statistical significance was determined using a paired two-tailed t-test ($p < 0.05$, $p < 0.01$).

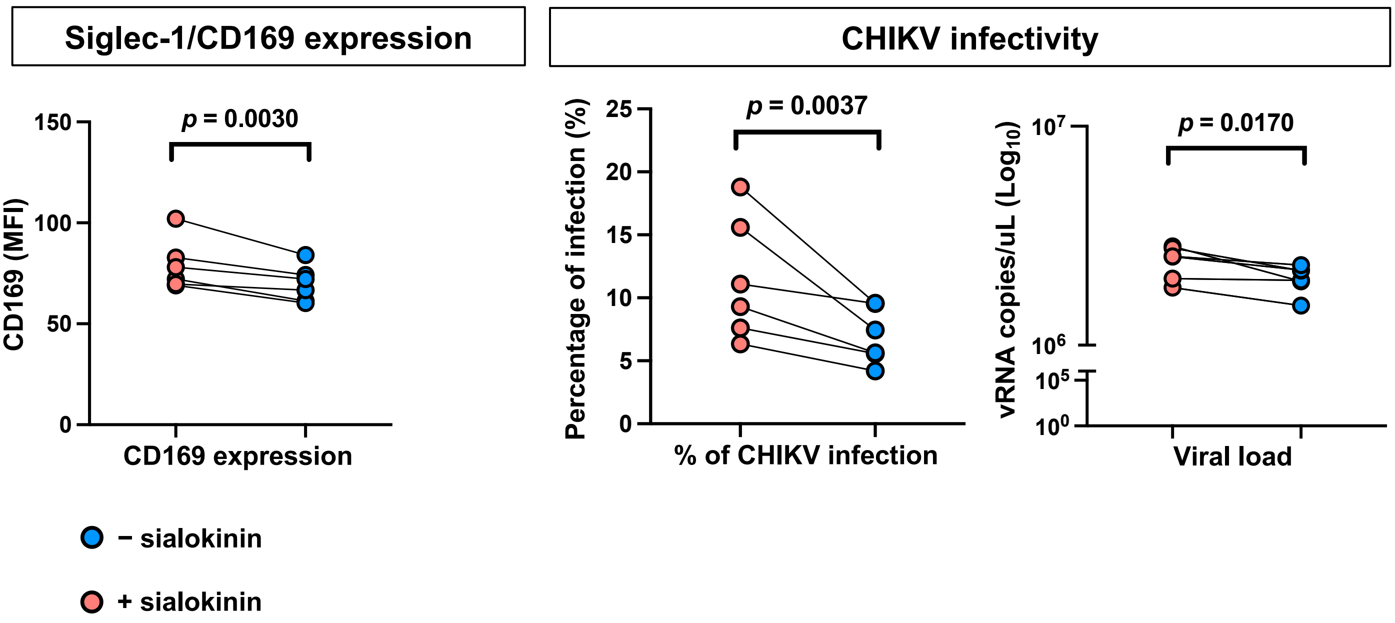

Supplementary Fig. 8: Pre-treatment of human monocytes with sialokinin reduces CHIKV infection and CD169 expression at 24 hours post-infection. Human monocytes isolated from healthy donors were pre-treated with sialokinin (10 µM) for 1 hour prior to infection with CHIKV (MOI = 10). At 24 hours post-infection, cells were analyzed by flow cytometry to assess activation profiles and infection levels. Pre-treatment with sialokinin resulted in a marked reduction in CD169 surface expression and CHIKV infectivity compared to untreated controls. These findings suggest that sialokinin modulates monocyte susceptibility to CHIKV by dampening both viral infection and activation marker expression. *$p < 0.05$, **$p < 0.01$ by paired two-tailed t-test.

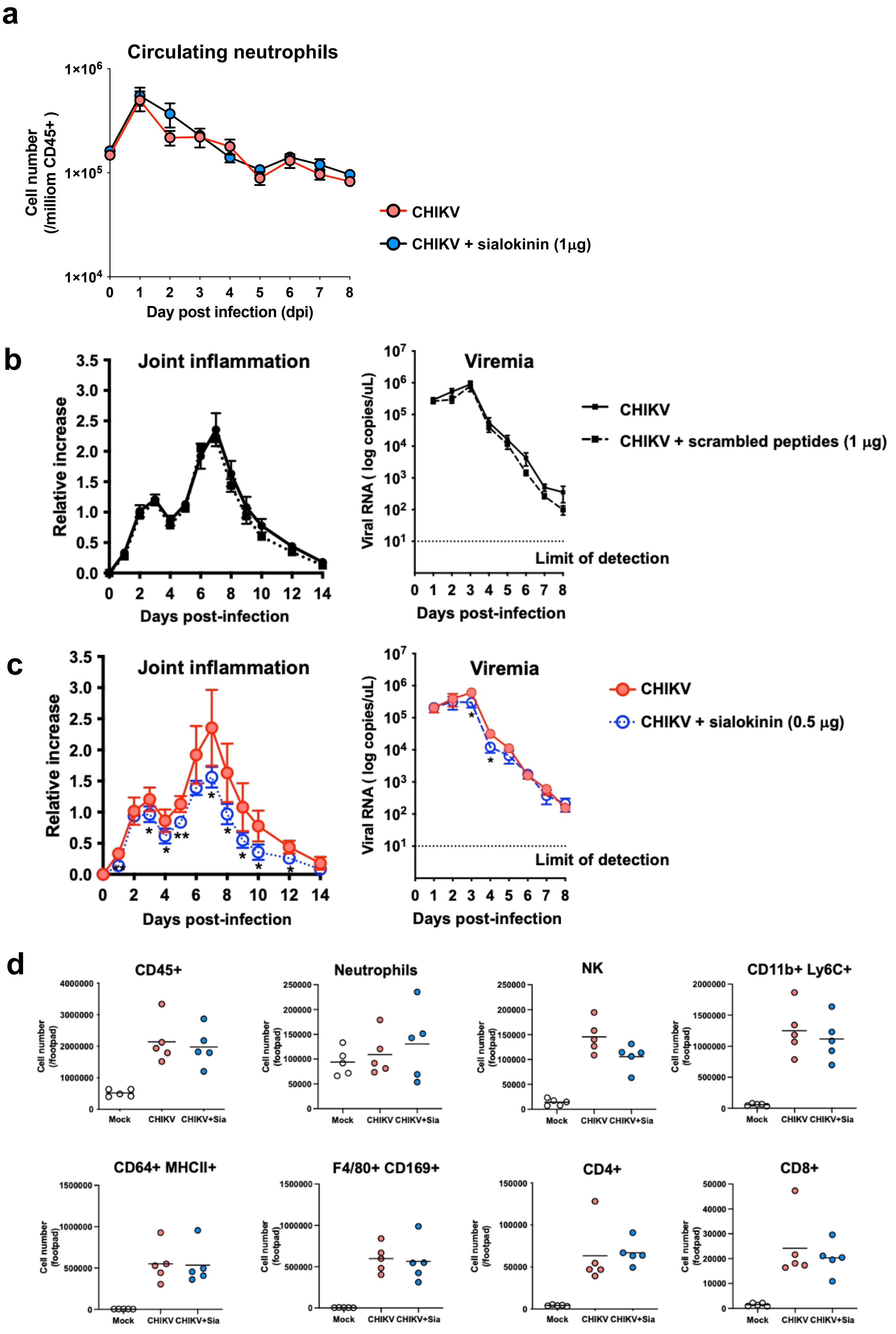

a
Circulating neutrophils
Cell number (/milliom CD45+)
1×10^6
1×10^5
1×10^4
0 1 2 3 4 5 6 7 8
Day post infection (dpi)
CHIKV
CHIKV + sialokinin (1μg)
b
Joint inflammation
Relative increase
3.5 3.0 2.5 2.0 1.5 1.0 0.5 0.0
0 2 4 6 8 10 12 14
Days post-infection
Viremia
Viral RNA (log copies/uL)
10^7 10^6 10^5 10^4 10^3 10^2 10^1
Limit of detection
0 1 2 3 4 5 6 7 8
Days post-infection
CHIKV
CHIKV + scrambled peptides (1 μg)
c
Joint inflammation
Relative increase
3.5 3.0 2.5 2.0 1.5 1.0 0.5 0.0
0 2 4 6 8 10 12 14
Days post-infection
Viremia
Viral RNA (log copies/uL)
10^7 10^6 10^5 10^4 10^3 10^2 10^1
Limit of detection
0 1 2 3 4 5 6 7 8
Days post-infection
CHIKV
CHIKV + sialokinin (0.5 μg)
d
CD45+
Cell number (/footpad)
4000000 3000000 2000000 1000000 0
Mock CHIKV CHIKV+Sia
Neutrophils
Cell number (footpad)
250000 200000 150000 100000 50000 0
Mock CHIKV CHIKV+Sia
NK
Cell number (/footpad)
250000 200000 150000 100000 50000 0
Mock CHIKV CHIKV+Sia
CD11b+ Ly6C+
Cell number (footpad)
2000000 1500000 1000000 500000 0
Mock CHIKV CHIKV+Sia
CD64+ MHCII+
Cell number (footpad)
1500000 1000000 500000 0
Mock CHIKV CHIKV+Sia
F4/80+ CD169+
Cell number (footpad)
1500000 1000000 500000 0
Mock CHIKV CHIKV+Sia
CD4+
Cell number (/footpad)
150000 100000 50000 0
Mock CHIKV CHIKV+Sia
CD8+
Cell number (footpad)
50000 40000 30000 20000 10000 0
Mock CHIKV CHIKV+Sia

Supplementary Fig. 9: Effect of sialokinin treatment in CHIKV-infected mice. a, C57BL/6 wild-type (WT) 4-week-old mice (CHIKV, n = 8; CHIKV + sialokinin, n = 10) were infected with 1 $\times$ 10^6 PFU of CHIKV via subcutaneous injection into the joint footpad. Sialokinin-treated mice received 1 µg of sialokinin simultaneously with CHIKV. $CD11b^+Ly6G^+$ neutrophils in blood were monitored daily by flow cytometry. Data from two independent experiments are presented as mean $\pm$ SEM. Statistical analysis was performed using the two-tailed Mann–Whitney U test. **b,** C57BL/6 WT 4-week-old mice [CHIKV, n = 10; CHIKV + scrambled peptide (1 μg), n = 10] were infected with 1x 10^6 pfu of CHIKV subcutaneously at the joint footpad. Joint swelling was monitored over 14 days post-infection (dpi). Viremia was assessed from tail vein blood (1 to 8 dpi) via qRT-PCR targeting CHIKV nsP1. Data from two independent experiments are shown as mean $\pm$ SEM. $^*p < 0.05$, $^{**}p < 0.01$, $^{***}p < 0.001$ by two-tailed *t*-test. **c,** Dose response of sialokinin in CHIKV-infected mice [CHIKV, n = 10; CHIKV + sialokinin (1 μg), n = 10; CHIKV + sialokinin (0.5 μg), n = 10)]. Mice were infected with 1x 10^6 pfu of CHIKV subcutaneously at the joint footpad. For the sialokinin groups, the mice were infected with CHIKV and given sialokinin simultaneously. Joint swelling was monitored to 14 dpi, viremia was assessed from the tail vein blood from 1 to 8 dpi, and viral load was quantified by qRT-PCR targeting CHIKV nsP1. Data from two independent experiments are shown as mean $\pm$ SEM. $^*p < 0.05$, $^{**}p < 0.01$, $^{***}p < 0.001$ by two-tailed *t*-test. **d,** Cellular profiles in the joint footpads were analyzed at 6dpi (CHIKV, n = 5; CHIKV + sialokinin 1 μg, n=5; and mock, n=5) mice at 6 dpi. Data are presented as mean $\pm$ S and analyzed using one-way ANOVA with Tukey's post-hoc test.

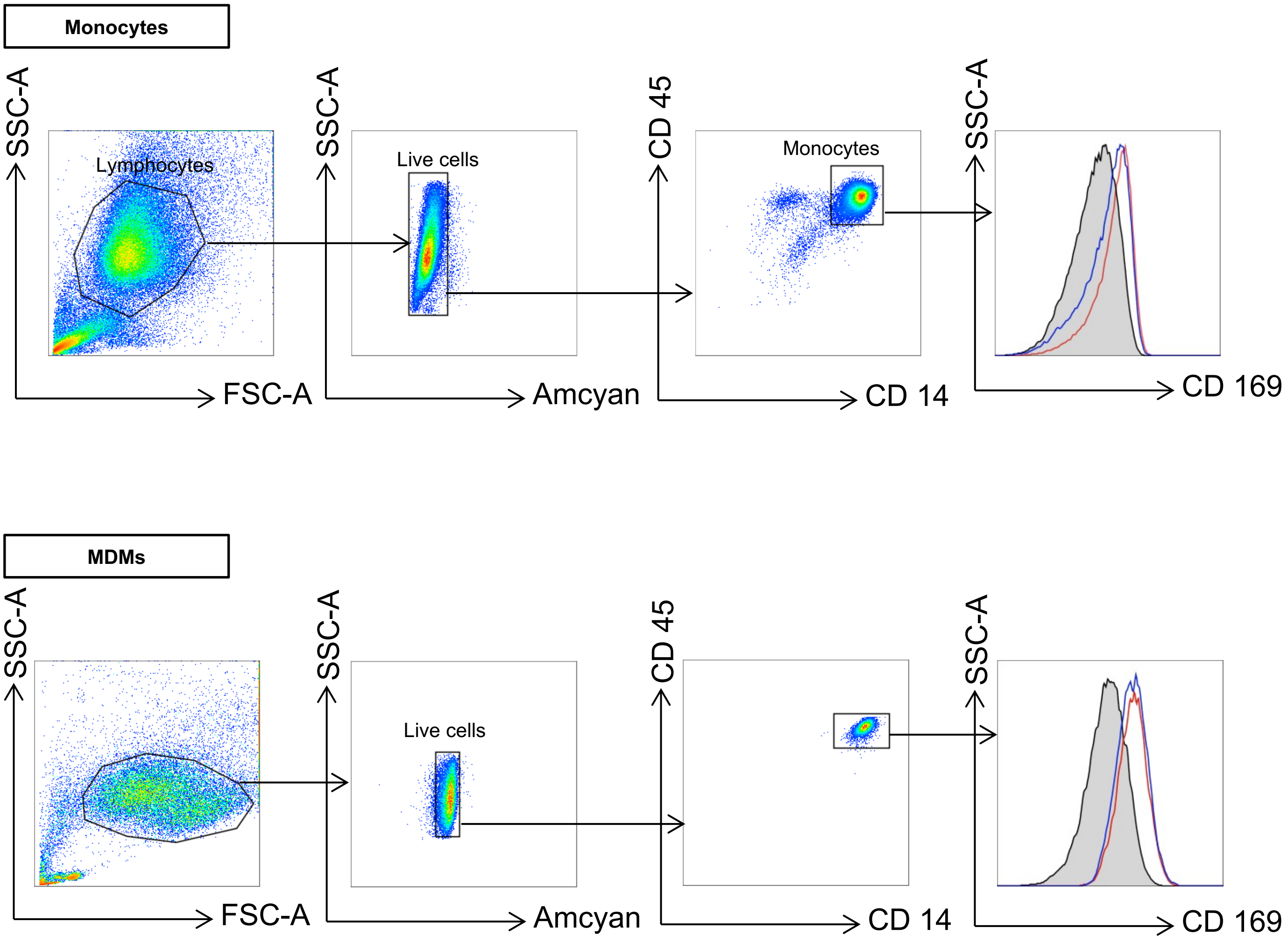

Supplementary Fig. 10: Representative flow cytometry gating strategy for human primary monocytes and monocyte-derived macrophages (MDMs). The cells were stained with a panel of antibodies to assess activation status of the cells following treatment/ CHIKV infection. Gating was performed in FlowJo as presented in this figure.

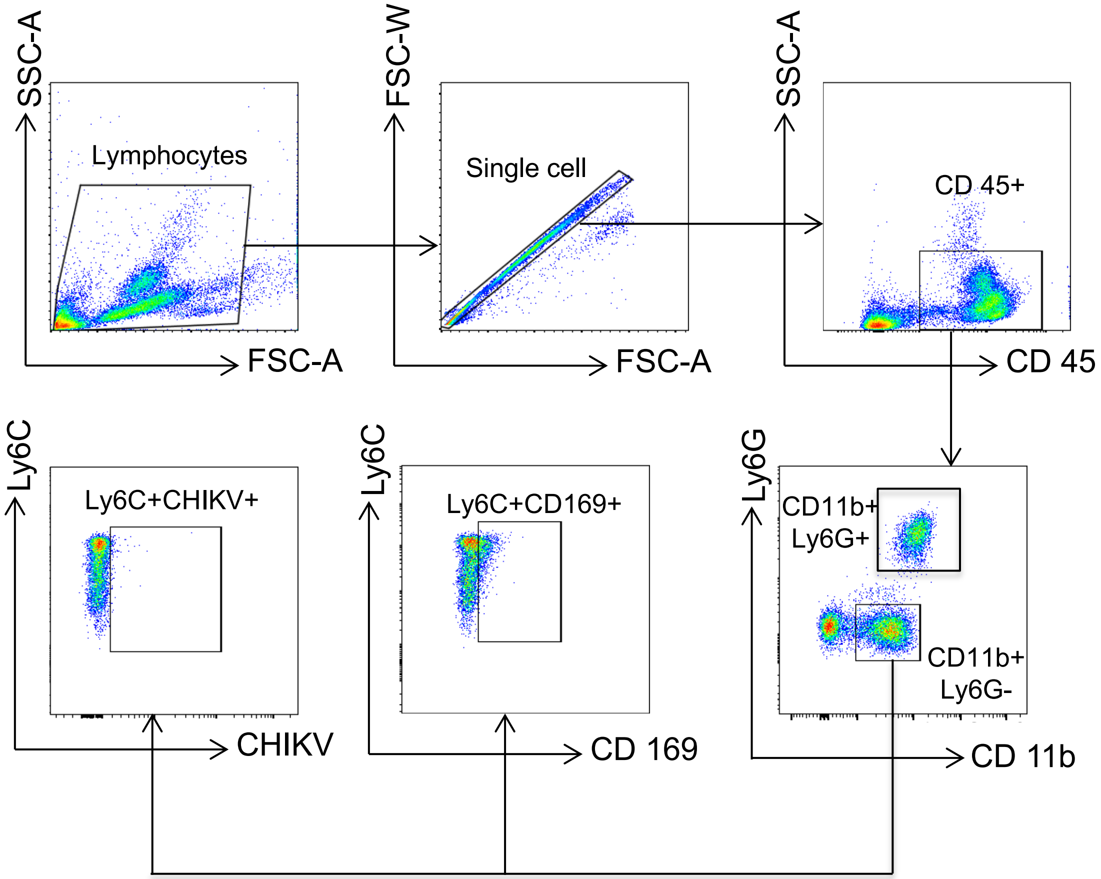

Supplementary Fig. 11: Representative flow cytometry gating strategy for circulating neutrophils and monocytes in CHIKV-infected C57BL/6 wild-type mice. Whole blood samples were stained with a panel of antibodies to profile the composition of immune cell subsets in the blood circulation. Gating was performed in FlowJo as presented in this figure.

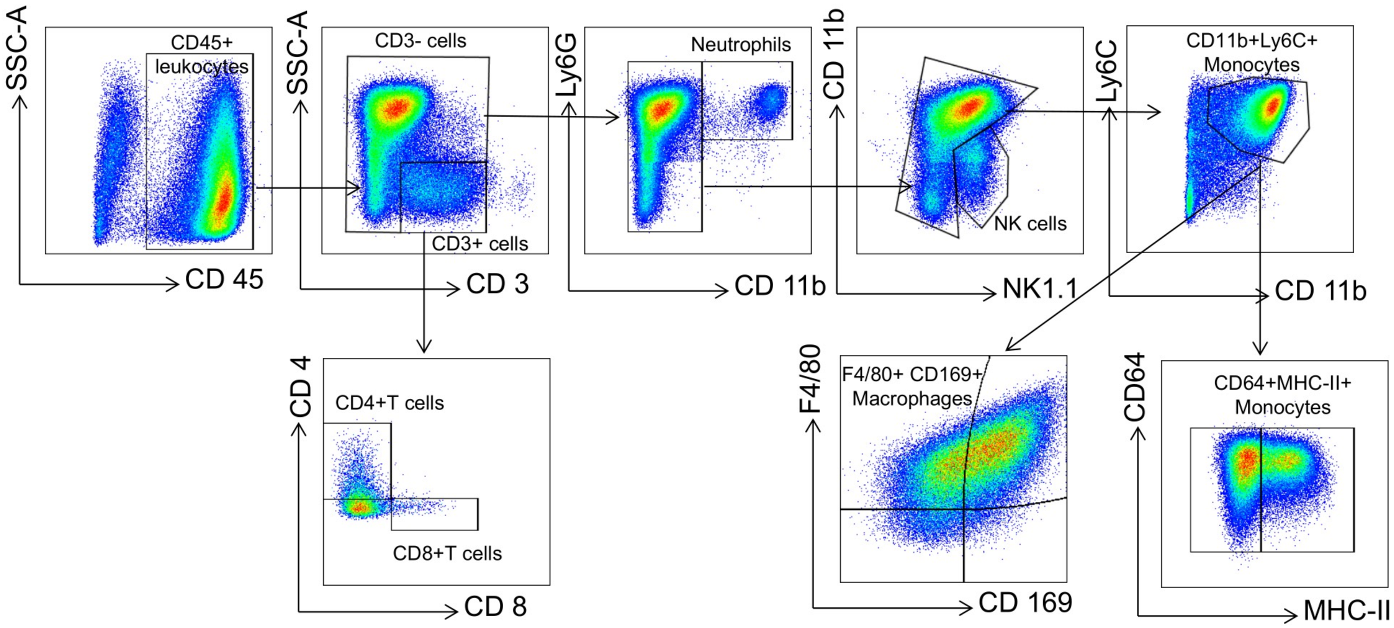

Supplementary Fig. 12: Representative flow cytometry gating strategy for neutrophils, monocytes, natural killer cells and T cell compartments. Isolated joint cells were stained with a panel of antibodies to profile the composition of immune cell subsets at the mouse joint footpad. Gating was performed in FlowJo as presented in this figure.
