# Supplementary material for: Mosquito salivary sialokinin reduces monocyte activation and chikungunya virus-induced inflammation via neurokinin receptors
Source: Nat Commun. 2025 Oct 20;16:8644. doi: 10.1038/s41467-025-64468-x (PMC12537910; doi:10.1038/s41467-025-64468-x)
Supplement: Supplementary file 2 — Reporting Summary [file 41467_2025_64468_MOESM2_ESM.pdf]

nature portfolio

Corresponding author(s): Siew-Wai Fong

Last updated by author(s): 12/7/2025

Reporting Summary

Nature Portfolio wishes to improve the reproducibility of the work that we publish. This form provides structure for consistency and transparency in reporting. For further information on Nature Portfolio policies, see our Editorial Policies and the Editorial Policy Checklist.

Statistics

For all statistical analyses, confirm that the following items are present in the figure legend, table legend, main text, or Methods section.

n/a	Confirmed	
☐	☒	The exact sample size (*n*) for each experimental group/condition, given as a discrete number and unit of measurement
☐	☒	A statement on whether measurements were taken from distinct samples or whether the same sample was measured repeatedly
☐	☒	The statistical test(s) used AND whether they are one- or two-sided<br>*Only common tests should be described solely by name; describe more complex techniques in the Methods section.*
☒	☐	A description of all covariates tested
☐	☒	A description of any assumptions or corrections, such as tests of normality and adjustment for multiple comparisons
☐	☒	A full description of the statistical parameters including central tendency (e.g. means) or other basic estimates (e.g. regression coefficient) AND variation (e.g. standard deviation) or associated estimates of uncertainty (e.g. confidence intervals)
☐	☒	For null hypothesis testing, the test statistic (e.g. *F*, *t*, *r*) with confidence intervals, effect sizes, degrees of freedom and *P* value noted<br>*Give P values as exact values whenever suitable.*
☒	☐	For Bayesian analysis, information on the choice of priors and Markov chain Monte Carlo settings
☒	☐	For hierarchical and complex designs, identification of the appropriate level for tests and full reporting of outcomes
☐	☒	Estimates of effect sizes (e.g. Cohen's *d*, Pearson's *r*), indicating how they were calculated

Our web collection on statistics for biologists contains articles on many of the points above.

Software and code

Policy information about availability of computer code

Data collection	MinKNOW 1.11.5, Guppy GPU base caller to collect RNAseq data
Data analysis	STAR aligner, featureCounts, edgeR Bioconductor package for RNAseq data analysis. Statistical analyses were performed using GraphPad Prism (version 9.0.0; GraphPad Software). Parametric or non-parametric tests were used to compare between groups and controls, as appropriate. p values less than 0.05 were considered statistically significant. Plots were generated using GraphPad Prism version 9.0.0.

For manuscripts utilizing custom algorithms or software that are central to the research but not yet described in published literature, software must be made available to editors and reviewers. We strongly encourage code deposition in a community repository (e.g. GitHub). See the Nature Portfolio guidelines for submitting code & software for further information.

Data

Policy information about availability of data

All manuscripts must include a data availability statement. This statement should provide the following information, where applicable:

- Accession codes, unique identifiers, or web links for publicly available datasets
- A description of any restrictions on data availability
- For clinical datasets or third party data, please ensure that the statement adheres to our policy

Data from RNAseq using Nanopore Technology have been deposited to NCBI GEO (GSE291153)

Research involving human participants, their data, or biological material

Policy information about studies with human participants or human data. See also policy information about sex, gender (identity/presentation), and sexual orientation and race, ethnicity and racism.

Reporting on sex and gender	clinical data used for association and correlation analysis were obtained from published datasets deposited by the cohort. Citation of the dataset is included in the manuscript.
Reporting on race, ethnicity, or other socially relevant groupings	clinical data used for association and correlation analysis were obtained from published datasets deposited by the cohort. Citation of the dataset is included in the manuscript.
Population characteristics	clinical data used for association and correlation analysis were obtained from published datasets deposited by the cohort. Citation of the dataset is included in the manuscript.
Recruitment	clinical data were obtained from published datasets deposited by the cohort. Citation of the dataset is included in the manuscript.
Ethics oversight	The study was approved by the National Healthcare Group's domain-specific ethics review board (DSRB No. B/08/026).

Note that full information on the approval of the study protocol must also be provided in the manuscript.

Field-specific reporting

Please select the one below that is the best fit for your research. If you are not sure, read the appropriate sections before making your selection.

☒ Life sciences ☐ Behavioural & social sciences ☐ Ecological, evolutionary & environmental sciences

For a reference copy of the document with all sections, see nature.com/documents/nr-reporting-summary-flat.pdf

Life sciences study design

All studies must disclose on these points even when the disclosure is negative.

Sample size	Sample size for each experiment is included in the figure legend.
Data exclusions	No exclusion
Replication	Key experiments were replicated at least twice using identical protocols.
Randomization	Animals were randomly allocated to different groups,
Blinding	NA

Reporting for specific materials, systems and methods

We require information from authors about some types of materials, experimental systems and methods used in many studies. Here, indicate whether each material, system or method listed is relevant to your study. If you are not sure if a list item applies to your research, read the appropriate section before selecting a response.

Materials & experimental systems

n/a	Involved in the study	
☐	☒	Antibodies
☐	☒	Eukaryotic cell lines
☒	☐	Palaeontology and archaeology
☐	☒	Animals and other organisms
☐	☒	Clinical data
☒	☐	Dual use research of concern
☒	☐	Plants

Methods

n/a	Involved in the study	
☒	☐	ChIP-seq
☐	☒	Flow cytometry
☒	☐	MRI-based neuroimaging

Antibodies

Antibodies used	BUV395-conjugated anti-mouse CD45 (clone 30-F11; BD

Antibodies used	Biosciences), Pacific Blue-conjugated anti-mouse CD4 (clone RM4-5; BioLegend), CF594-conjugated anti-mouse CD8 (clone 53-6.7; BD Biosciences), PE-Cy7-conjugated anti-mouse CD3 (clone 17A2; BioLegend), APC-Cy7-conjugated anti-mouse Ly6C (clone HK1.4; BioLegend), Alexa Fluor 700-conjugated anti-mouse MHC-II (clone M5/114.15.2; BioLegend), PerCPCy5.5-conjugated anti-mouse LFA-1 (clone H155-78; BioLegend), BV650-conjugated anti-mouse CD11b (clone M1/70; BioLegend), BV605-conjugated anti-mouse CD11c (clone N418; BioLegend), CF594-conjugated anti-mouse Ly6G (clone 1A8; BD Biosciences), eFluor450-conjugated anti-mouse B220 (clone RA3-6B2; eBioscience), PE-conjugated anti-mouse MerTK (clone DS5MMER; eBioscience), APC-conjugated anti-mouse CD64 (clone X54-5/7.1; BioLegend), biotin-conjugated anti-mouse NK1.1 (clone PK136; eBioscience), and BUV737 streptavidin (BD Biosciences).P-PI3K (p85; Tyr458/p55; Tyr199, Cell Signaling Technology, 1:2000), PI3K (p85, Cell Signaling Technology, 1:2000), P-Akt (Ser473, Cell Signaling Technology, 1:2000), and Akt (Cell Signaling Technology, 1:2000).All antibodies used in ex vivo human monocytes and MDMs experiments were mouse anti-human antibodies and were obtained from BD Pharmingen (CD3, CD19, CD20, CD14, CD69, CD56, CD94, NKG2D, CD107a, and IFN-γ), BioLegend (CD16 and CD45), or Miltenyi Biotec (NKG2A)
Validation	The commercial antibodies used for flow-cytometry, western blot,ELISA., were validatedby the successful separation of the given positive and negative populations, as well as by referring to the the validation statementsf rom manufactures.

Eukaryotic cell lines

Policy information about cell lines and Sex and Gender in Research

Cell line source(s)	Chinese hamster ovary (CHO) cells expressing three distinct types of human NK receptors (NK1R, NK2R, and NK3R) were a generous gift from Dr Shinya Oishi, Faculty of Pharmaceutical Sciences, Kyoto University, Japan. Vero-e6 cells and C6/36 cells used for virus propagation were obtained commercially.
Authentication	NA
Mycoplasma contamination	All cells were tested free of Mycoplasma contamination.
Commonly misidentified lines (See ICLAC register)	No commonly misidentified lines were used.

Animals and other research organisms

Policy information about studies involving animals; ARRIVE guidelines recommended for reporting animal research, and Sex and Gender in Research

Laboratory animals	C57BL/6 WT 4-week-old mice were used for CHIKV infection
Wild animals	No wild animals were used.
Reporting on sex	All the C57BL/6 WT mice used were female mice
Field-collected samples	No field-collected samples were used.
Ethics oversight	All the experimental procedures were approved by the local ethical committee

Note that full information on the approval of the study protocol must also be provided in the manuscript.

Clinical data

Policy information about clinical studies

All manuscripts should comply with the ICMJE guidelines for publication of clinical research and a completed CONSORT checklist must be included with all submissions.

Clinical trial registration	NA
Study protocol	The study was conducted with approved study protocol. The protocol was was approved by the National Healthcare Group's domain-specific ethics review board (DSRB No. B/08/026).
Data collection	Data was collected with approved study protocol. The protocol was was approved by the National Healthcare Group's domain-specific ethics review board (DSRB No. B/08/026).
Outcomes	Outcomes of the study was published and deposited. Citation of the dataset is included in the manuscript.

Plants

Seed stocks

NA

Novel plant genotypes

NA

Authentication

NA

Flow Cytometry

Plots

Confirm that:

☒ The axis labels state the marker and fluorochrome used (e.g. CD4-FITC).

☒ The axis scales are clearly visible. Include numbers along axes only for bottom left plot of group (a 'group' is an analysis of identical markers).

☒ All plots are contour plots with outliers or pseudocolor plots.

☒ A numerical value for number of cells or percentage (with statistics) is provided.

Methodology

Sample preparation

Following harvesting, cells were specifically stained for the surface markers CD45 and CD14 (for CHIKV-infected monocytes and MDMs). Dead cells were excluded by staining with the LIVE/DEAD Fixable Aqua Dead Cell Stain Kit (Life Technologies). The stained cells were subsequently incubated with FACS lysing solution (BD Biosciences) to lyse the red blood cells. All antibodies used were mouse anti-human antibodies and were obtained from BD Pharmingen (CD3, CD19, CD20, CD14, CD69, CD56, CD94, NKG2D, CD107a, and IFN-γ), BioLegend (CD16 and CD45), or Miltenyi Biotec (NKG2A). Data were acquired on a Fortessa flow cytometer (BD Biosciences) with BD FACSDiva software version 9.0. Data analysis was performed using FlowJo version 9.3.2 (Tree Star, Inc.).Isolated joint cells were first blocked with 1% mouse/rat serum (Sigma-Aldrich) blocking buffer for 10 min. The cells were then stained for 20 min with the following antibodies: BUV395-conjugated anti-mouse CD45 (clone 30-F11; BD Biosciences), Pacific Blue-conjugated anti-mouse CD4 (clone RM4-5; BioLegend), CF594-conjugated anti-mouse CD8 (clone 53-6.7; BD Biosciences), PE-Cy7-conjugated anti-mouse CD3 (clone 17A2; BioLegend), APC-Cy7-conjugated anti-mouse Ly6C (clone HK1.4; BioLegend), Alexa Fluor 700-conjugated anti-mouse MHC-II (clone M5/114.15.2; BioLegend), PerCPCy5.5-conjugated anti-mouse LFA-1 (clone H155-78; BioLegend), BV650-conjugated anti-mouse CD11b (clone M1/70; BioLegend), BV605-conjugated anti-mouse CD11c (clone N418; BioLegend), CF594-conjugated anti-mouse Ly6G (clone 1A8; BD Biosciences), eFluor450-conjugated anti-mouse B220 (clone RA3-6B2; eBioscience), PE-conjugated anti-mouse MerTK (clone DS5MMER; eBioscience), APC-conjugated anti-mouse CD64 (clone X54-5/7.1; BioLegend), biotin-conjugated anti-mouse NK1.1 (clone PK136; eBioscience), and BUV737 streptavidin (BD Biosciences). Samples were acquired on a LSR II flow cytometer (BD Biosciences) with FACSDiva software and analysed using FlowJo software.

Instrument

Fortessa flow cytometer (BD Biosciences) and LSR II flow cytometer (BD Biosciences)

Software

BD FACSDiva software version 9.0 and FlowJo version 9.3.2 (Tree Star, Inc.).

Cell population abundance

No sorting carried out

Gating strategy

Single cells were gated based on FSC-A and SSC-A features. Live cells were gated based on Live/Dead negative staining.

Gating strategy	Boundaries of of the gates were determined by by the visualization of of distinct populations or or comparing samples and positive/negative controls.

☒ Tick this box to confirm that a figure exemplifying the gating strategy is provided in the Supplementary Information.
